# Is a career in industry for you?

**DOI:** 10.1063/4.0001089

**Published:** 2025-10-27

**Authors:** Pamela A Williams

**Affiliations:** Astex Pharmaceuticals, Cambridge, Cambs, United Kingdom

## Abstract

Pamela Williams is a structural biologist with more than twenty years of experience working in a pharmaceutical setting, supporting medicinal chemistry campaigns with protein-ligand structures, from initial fragments to final lead compounds. Following a bachelor’s degree in mathematics she was awarded a DPhil in time-resolved studies on macromolecules from the Laboratory of Molecular Biophysics, University of Oxford. She continued her postdoctoral research at the at The Scripps Research Institute, La Jolla, where she worked on the X-ray structure determination of metalloenzymes involved in electron transfer and xenobiotic metabolism. She then returned to the UK to continue working on the structure determination of enzymes involved in metabolism at Astex Pharmaceuticals. In 2016 Pamela lead the evaluation of cryoEM as an emerging structural technique for structure-based drug discovery, and her current focus is the industrialisation of cryoEM workflows that will allow the full impact of cryoEM on the development on drugs to be realised.

